# Unveiling the determinants of femicide in Ecuador: a comprehensive analysis

**DOI:** 10.3389/fsoc.2024.1452438

**Published:** 2025-01-03

**Authors:** Juan Pablo Díaz-Sánchez, Cintya Lanchimba, Diego Albuja, Yulissa Ramírez

**Affiliations:** Escuela Politécnica Nacional, Quito, Ecuador

**Keywords:** femicide, gender-based violence, determinants, Ecuador, policy implications

## Abstract

This paper reports an investigation of the determinants of femicide using the context of Ecuador as case of study. To do so, we use official data spanning 2018 to 2022 from the National Survey on Family Relations and Gender Violence against Women in Ecuador with a dataset revealing 1,325 femicides and violent deaths of cisgender women and girls. Using a probit regression model, we find that several variables related to perpetrators’ such as level of education, employment status, and variables related to the crime itself such as location emerge as pivotal factors in understanding femicide incidents. This study contributes to a more profound comprehension of femicide’s multifaceted determinants, emphasizing the dynamic nature of these factors. The research aids in the development of evidence-based policies to address this societal issue effectively.

## 1 Introduction

Femicide, as a harrowing manifestation of gender-based violence, constitutes a critical issue transcending the boundaries of social concerns (Dayan, 2021). This research has been motivated by the quest to delve into the determinants and factors underlying femicide within the unique context of Ecuador’s developing economy. The importance of this study is underscored by the gravity of the issue: femicide represents not only a crime but also an economic burden, prompting the urgent need for comprehensive research and evidence-based policymaking.

The worldwide recognition of the imperative to eliminate all forms of violence against women and girls, as encapsulated in the United Nations’ Sustainable Development Goals (United Nations, 2022), cannot be overstated. Despite this global recognition and the continuous public-private collaboration to end this gender-based violence, one in three women globally, amounting to 736 million individuals, has been a victim of physical and/or sexual violence (United Nations, 2022).

Moreover, the emergence of the COVID-19 pandemic has cast a disconcerting shadow, exacerbating the prevalence of violence against women (Lanchimba et al., 2020). Femicide, the ultimate manifestation of violence against women, represents the stark culmination of abuse and brutality, underlining the dire need for a comprehensive national policy to prevent and combat it (Dawson and Gartner, 1998).

The existing literature on femicide elucidates numerous causal factors, and it is imperative to consider the insights provided by scholars such as Sharps et al. (2001). Their work highlights the distressing reality that many women experience prior violence before femicide occurs, signifying that such violence may be preventable. These precursory violent acts may include sexual assault by intimate partners, a distressing form of aggression that is often underreported (McFarlane et al., 2005).

However, in many societies, there remains limited information about individuals seeking psychological or any form of assistance regarding violence, due to the persisting taboos and normalization of certain abusive behaviors (Cullen et al., 2021; Lanchimba et al., 2023). Gender inequality further exacerbates this issue, perpetuating violence and potentially creating an environment conducive to femicide. Women may not only face restricted access to information, healthcare, or support services, but also have less control over how or whether they can utilize these resources, even when they are available. This reduced autonomy undermines their ability to make decisions about their lives and safety, further increasing their vulnerability (Reckdenwald et al., 2018). Additionally, the broader socio-political structures often frame women as being valued less than men or even as men’s property, a perception that fuels acts of femicide where women are treated as expendable objects (Radford and Russell, 1992). Such gendered power imbalances lay the groundwork for systemic violence, culminating in the ultimate manifestation of control and devaluation—femicide.

The extent of femicide, beginning with violence against women and girls, is deeply rooted in the socio-political climate of how women are viewed and regarded across society. This systemic and structural representation of gender equity permeates various domains, from the home to school settings (including curriculum and teaching materials), workplaces, politics, and law. These societal structures and perceptions significantly influence the prevalence and normalization of violence against women, ultimately contributing to the occurrence of femicide.

This study, situated in the unique context of Ecuador, contributes to a more comprehensive understanding of these multifaceted issues. We employed a probit model, with the dependent variable indicating whether a femicide was committed or not, using data derived from the inaugural National Survey on Family Relations and Gender Violence against Women in Ecuador (ENVIGMU), conducted by the National Institute of Statistics and Census (INEC) in 2011 and Consejo de la Judicatura in 2022. This dataset exposes a toll of 1,325 femicides and violent deaths of women over the 2014–2022 period.

To provide a structured overview, this paper is organized as follows. Section 2 presents a comprehensive literature review of femicide and the factors that increase its incidence. Section 3 introduces the methods, including our dataset. The methodological approach is outlined in Section 4. Section 5 presents our research findings. Finally, Sections 6 and 7 offer a discussion and concluding remarks, respectively.

## 2 Literature review

The literature review in this paper delves into the multifaceted concept of femicide, offering a comprehensive understanding of its definition and the various drivers associated with this tragic phenomenon. Despite its complexity, most social scientists concur that femicide is the murder of women and girls simply because of their gender. Our analysis relies on this definition, but it primarily focuses on Ecuador’s legal definition of femicide, which emphasizes the relationship between the victims and their perpetrators. To provide a solid foundation for our empirical study, we draw from a wide array of scholarly sources and theories to explore the intricacies of this concern.

### 2.1 Defining femicide: a comprehensive examination

Femicide, defined as the killing of women because of their gender, is rooted in historical gender-based violence and inequality. Radford and Russell (1992) first introduced the term, emphasizing that gender motivation is key to distinguishing femicide from other homicides. However, debates continue over whether the sex of the victim alone suffices, or if explicit gender motivation must be present (Weil and Keshet, 2021). The socio-ecological model helps frame these debates by examining factors at individual, relational, and societal levels that contribute to gender-based violence.

At the individual level, the victim’s and perpetrator’s sex are central, but relational dynamics often play a pivotal role. Many femicides occur within intimate partner relationships, where prolonged abuse, control, and subordination lead to fatal violence (Toprak and Ersoy, 2017). This dynamic, where women have reduced autonomy and control over resources, exacerbates their vulnerability to violence. Relational factors are critical in understanding femicide, as these power imbalances are key determinants.

On a broader scale, societal structures, including gender inequality and cultural norms, perpetuate violence against women. Studies highlight the systemic devaluation of women, framing them as property or less valuable than men, which fuels gender-motivated killings (Radford and Russell, 1992; Walby et al., 2017). These societal factors, combined with limited data on other key dimensions like economic or community contexts, underscore the need for multi-level approaches to fully understand and combat femicide.

### 2.2 Legal definition

The adoption of femicide in the legal systems across the world has been a gradual process with different approaches. Despite its common elements, the legal definition of femicide varies across countries. A pivotal moment in Ecuador’s legal framework occurred in 2014 when femicide was formally defined as a specific type of crime within the nation’s criminal law. On August 10th, 2014, the enactment of the new Código Integral Penal (COIP) delineated femicide as the murder of women fueled by hatred for their gender, punishable by a prison sentence of up to 26 years (COIP, 2014, art. 141). Furthermore, article 142 of this criminal law recognizes a close relationship between the victim and the perpetrator as an aggravating circumstance.

A report from the Attorney General’s office (2016) suggests that while judges and prosecutors can use a gender approach to determine the motive of the murder of a woman, in most cases, a close relationship between the victim and the perpetrator is interpreted as a power struggle and constitutes a determinant factor in distinguishing murder from femicide. Therefore, the definition of femicide used in this analysis is closer to the view of Stout (1992) who defines this crime as the murder of a woman by their intimate partner.

Given our data comes from official sources and relies on the legal definition of femicide, our analysis will mainly focus on the relationship between the victim and the perpetrator. While determining the motive of a perpetrator to kill a woman would be ideal to distinguish murder from femicide, we recognize the difficulty of this task as well as the limitations of collecting reliable information on this dimension.

### 2.3 Factors associated with the incidence of femicide

An extensive body of literature analyzes the factors that affect the incidence of femicide from a qualitative perspective (García-Vergara et al., 2022; Decker et al., 2004; Emerson Dobash and Dobash, 2011; Sebire, 2017; Monckton Smith, 2020; David and Jaffe, 2021). However, few studies analyze this phenomenon using a quantitative approach. Stratus (1994), for example, employs a multiple regression analysis to measure the effect of several factors like gender inequality or income inequality on intimate partner assault from a social perspective. Within this framework, Frye and Wilt (2001) employ the social disorganization theory to explore how prohibited behaviors within social groups impact rates of both femicide and homicide.

Education plays a central role in shaping societal attitudes toward gender-based violence and is essential in addressing gender inequity, gender stereotyping, and gender bias, which are foundational to tackling the extreme manifestation of such biases: femicide. According to Subrahmanian (2005), educational settings provide a critical platform for challenging entrenched gender norms and promoting gender equality. Thus, Schools can play a pivotal role in preventing gender-based violence.

Additionally, the cultural context of machismo in South and Central American nations, including Ecuador, cannot be ignored when examining the determinants of femicide. Machismo, characterized by exaggerated masculine pride and dominance, often excuses and perpetuates violence against women (Stevens, 1965; Viveros-Vigoya, 2016). This cultural phenomenon contributes significantly to the normalization of violence and the subjugation of women, reinforcing the power dynamics that lead to femicide. Recent research endeavors have expanded the scope of analysis, recognizing the significance of structural conditions affecting both victims and aggressors in femicide cases. McCall et al. (2010), Corradi et al. (2016), and Grosfoguel et al. (2015) emphasize the role of race, socioeconomic status, and nationality as axes of social domination contributing to femicide. Furthermore, Pinchevsky and Wright (2012) highlight the adverse impact of low education levels on femicide rates, elucidating that lower educational attainment in potential aggressors increases the likelihood of femicide.

Age is another pivotal factor, as discussed by O’Campo et al. (1995) and Beyer et al. (2014). They stress that young women are often at a higher risk of becoming femicide victims, particularly those who marry early, interrupt their education, and become economically dependent on their partners.

Drawing parallels with the broader issue of homicide, Loftin and Hill (1974) argue that inequality, poverty, and social disorganization heighten the probability of femicide. They contend that women living in environments characterized by social disorganization and heightened criminal opportunity face an elevated risk of femicide, particularly when compared to those residing in more stable suburban areas (Grana, 2001).

While these studies have elucidated the factors associated with the incidence of femicide, Walby (2023) claims that most of the existing literature has had to adopt a pragmatic approach. For example, he explains that some analyses have aimed to implement a gender motivation approach but ended up using data on the sex of the victim because that is the only available. Similarly, García-Vergara et al. (2022) argue that most of the studies on violence against women focus on developed countries like the US or the UK due to the availability of information. Few studies focus on Latin America, and even fewer analyze the Ecuadorian case.

One is the work of Lanchimba et al. (2023), which explores the underlying dynamics influencing domestic violence. Similarly, Boira et al. (2017), highlight the prevalence of a chauvinistic culture and a deficient judicial system as the main factors that increased the incidence of femicide in Ecuador between 2011 and 2014. Along the same line, San Sebastián et al. (2021) explores the variables that shaped the concentration of femicide across this country in 2017. Yet, no study has explored the changes in this phenomenon during the last 4 years.

Recently, violence has become a concern among Ecuadorians. Police reports, for example, show that in the last 4 years, Ecuador has shifted from being one of the safest countries in Latin America to being one of the most violent. The homicide rate reached one hundred murders in two provinces and surpassed 60 victims in five in 2023. Similarly, as the result of our study will indicate, the incidence of femicides has also increased substantially; thus, turning Ecuador into a dangerous country even for women.

On the other hand, Ecuador’s geographical expanse spans a total area of 283,561 square kilometers (about half the area of Texas), ranking it as the fourth smallest country in the continent. However, its population density sets it apart, with a density of 68 inhabitants per square kilometer. This density has increased in recent years, attributed in part to the influx of migrating people, notably from Venezuela, as suggested by Díaz-Sánchez et al. (2020). This demographic shift has significant implications, both at the macro level, where population density is affected, and at the micro level, where household overcrowding becomes a pertinent concern.

Ecuador’s geography, demographics, recent legal developments, and the latest surge in violence led to its selection as the locus of our study on the drivers of femicide. The current research aims to conduct a quantitative analysis of the factors associated with the recent increase in violence against women by exploring the difference between femicides and other types of murders of women.

To summarize, this literature review has examined the multifaceted landscape of femicide. In the next section, we organize this into three distinct groups to structure our analysis: Group A focused on essential characteristics of both victims and perpetrators, emphasizing variables such as age, level of schooling, ethnicity, marital status, type of relationship, and profession. These characteristics have emerged as fundamental in unraveling the complexities of femicide, with insights suggesting that they significantly influence the dynamics of femicide cases.

Group B shifted the focus to the social context surrounding femicide, recognizing that this violence often thrives in environments marked by structural inequalities and social disorganization. We explored variables such as urban/rural residence and delved into the intricate relationship between femicide and broader societal factors, including economic disparities, systemic gender-based violence, and cultural norms.

Group C examined environmental factors and their influence on the probability of femicide. Notably, the frequency of crime in the victim’s environment emerged as a critical variable. This analysis underscored the interconnectedness of violence within communities and its role in elevating the risk of femicide.

## 3 Methods

As Table 1 exemplifies, the literature offers many potential factors that increase the likelihood of femicide. This marks the transition from literature review to methods, where we apply these findings to categorize the variables into three groups that will form the basis of our empirical analysis.

**Table 1:** Candidate determinants of femicide.

Determinant	Referred study
(A) Main characteristics	
(A1) Victim’s age and Perpetrator’s age	Beyer et al. (2014), Brewer and Smith (1995), and O’Campo et al. (1995)
(A2) Victim ethnic self-identification	Beyer et al. (2014) and Frye and Wilt (2001)
(A3) Victim’s civil status	McCall et al. (2010)
(A4) Victim’s profession	McCall et al. (2010)
(A5) Victim’s nationality and Perpetrator’s nationality	Beyer et al. (2014) and Frye and Wilt (2001)
(A6) Victim and Perpetrator’s level of education	Pinchevsky and Wright (2012)
(A7) Victim-aggressor relationship	Carcedo and Sagot (2000)
(B) Social disruption	
(B1) Inequality and poverty	Loftin and Hill (1974)
(B2) Area	Sepúlveda et al. (2018)
(C) Presence of risk factor	
(C1) Causes of death	Kouta et al. (2019) and Vignali et al. (2021)
(C2) Use of weapons (fire, sharp instruments)	Kouta et al. (2019) and Vignali et al. (2021)
(C3) Psychological disorder, drug abuse, drug trafficking	Sepúlveda et al. (2018) and Vignali et al. (2021)
(C4) Inadequate housing, overcrowding, economic dependence	Sepúlveda et al. (2018)
(C5) Reason for confrontation (argument, divorce, jealousy)	Karbeyaz et al. (2018)

Adapted and improved from the classification proposed by Vignali et al. (2021).

### 3.1 Group A: essential characteristics of both victims and perpetrators

Drawing from the foundational work of O’Campo et al. (1995), Beyer et al. (2014), and McCall et al. (2010). These characteristics include age, level of schooling, ethnicity, marital status, type of relationship, and profession. These features are fundamental in unraveling the complexities of femicide.

Age emerges as a critical factor, revealing the vulnerability of victims and the potential motivations of perpetrators (Lund et al., 2020). Insights from this literature suggest that both the age of victims and perpetrators significantly influence the dynamics of femicide cases (Hernández, 2021). Cunha et al. (2024) and Sebire (2017) concurs with this notion and argue that the age difference between the victim and her aggressor, particularly when the victim is younger, is strongly associated with the incidence of intimate partner femicide.

Level of schooling, as highlighted in Hernández (2021), stands as another essential variable. It serves as a lens to examine education’s impact on femicide rates. A lower level of education among potential aggressors may exacerbate the likelihood of femicide, emphasizing the significance of this variable in our analysis.

Ethnicity, a multifaceted aspect often intersecting with race, socioeconomic status, and nationality, is explored, aligning with (Shai et al., 2022). Understanding the ethnic backgrounds of victims and perpetrators allows for an examination of potential patterns or disparities within specific ethnic groups. Greenfield et al. (1998) claim that minorities are often at a higher risk of being victims of femicide. Recent studies confirm that racial minorities in the U.S. continue to experience higher rates of intimate partner femicide. According to the CDC’s National Intimate Partner and Sexual Violence Survey (2020), African American women remain disproportionately affected, with intimate partner violence being a leading cause of death among Black women aged 15–44. Marital status and the type of relationship shared between victims and perpetrators offer critical insights into the intimate nature of femicide. These variables illuminate the dynamics of abusive relationships, potentially shedding light on whether certain marital or relationship statuses are more susceptible to femicide incidents (Cullen et al., 2021; Dawson and Gartner, 1998).

Profession, as indicated by the research of Toprak and Ersoy (2017), Gould (2022), and Atuk and Craddock (2023) holds significance as it elucidates the role of occupation in femicide cases. By examining the occupational status of victims and perpetrators, we aim to identify potential correlations between certain professions and femicide rates, contributing to a nuanced analysis.

### 3.2 Group B: the social context surrounding femicide

Central to this examination is the recognition that femicide often thrives in environments marked by structural inequalities and social disorganization (Loftin and Hill, 1974; Swanson, 2013; Lund et al., 2020).

Accordingly, one pertinent variable includes an area indicator that distinguishes between urban and rural residences of victims. Existing literature emphasizes the role of place in influencing gender inequality and, consequently, the occurrence of femicide (Loftin and Hill, 1974; Swanson, 2013; Lund et al., 2020; Reckdenwald et al., 2018). Urban areas, characterized by higher population densities and increased exposure to various socio-economic disparities, may provide a distinct backdrop for femicide compared to rural settings (Swanson, 2013). On the other hand, other studies claim that the incidence of femicide and violence against women tend to be higher in rural areas (Reckdenwald et al., 2019).

Moreover, femicide is not an isolated act but is deeply intertwined with the broader social fabric (Morena et al., 2022; Richards et al., 2014; Shai et al., 2022). It is vital to consider factors such as economic disparity, systemic gender-based violence, and cultural norms that may create an environment conducive to femicide. Research by Sithomola (2020), Lund et al. (2020), and Hernández (2021) among others highlights the role of cultural and social factors in young adult intimate partner femicide, underscoring the significance of social norms and relationships within communities. Thus, the exploration of social context must encompass not only structural factors but also cultural and interpersonal dynamics.

Inequalities in access to resources and opportunities can exacerbate the risk of femicide. Some studies shed light on how children and their caregivers adjust after intimate partner femicide, revealing the profound impact of socio-economic disparities on the lives of survivors (Siriwardhane and Khan, 2021; Hernández, 2021; Reckdenwald et al., 2018; Hardesty et al., 2008). Understanding the repercussions of such disparities on victims and their families further emphasizes the need to contextualize femicide within the wider societal framework (Hernández, 2021).

Within the comprehensive landscape of femicide research, Group C emerges as a crucial focal point, shedding light on the pivotal role of the victim’s environment in influencing the probability of femicide. In this analysis, we draw insights from a selection of seminal studies by Karbeyaz et al. (2018), Kouta et al. (2019), Sepúlveda et al. (2018), Vignali et al. (2021), and Glass et al. (2008) to construct a compelling narrative regarding the significant risk factors associated with femicide.

### 3.3 Group C: environmental factors influencing femicide

Karbeyaz et al. (2018) provide valuable insights into the interconnectedness of violence within communities and femicide. Their research underscores that an environment characterized by high levels of criminality and violence can substantially elevate the risk of femicide (Cullen et al., 2019). This observation is particularly relevant in the context of the COVID-19 pandemic, which, as noted by Morena et al. (2022), exacerbated cases of violence and potentially escalated them to femicide.

The mental health impacts of the pandemic played a significant role, making it imperative to consider the broader societal context. Kouta et al. (2019), who emphasize the need to consider the broader societal context when addressing femicide, further support this perspective. They argue that the presence of crime in a victim’s environment serves as a significant red flag, requiring proactive interventions to prevent fatal outcomes. Proactive interventions are required to prevent fatal outcomes, especially in situations where mental health issues contribute to the escalation of violence and femicide, as was witnessed during the pandemic (Morena et al., 2022; Shai et al., 2022).

Sepúlveda et al. (2018) on the other hand, contribute to this discourse by highlighting the correlation between femicide rates and regions with a higher incidence of criminal activities. Their findings underscore the urgency of addressing not only the immediate circumstances but also the underlying factors that foster an environment conducive to femicide.

Furthermore, Vignali et al. (2021) offers a nuanced perspective by incorporating the cause of death variable into the empirical model. This inclusion allows for a more in-depth exploration of how crime within a victim’s environment directly correlates with the likelihood of femicide. Their research alongside the work of Glass et al. (2008) exemplify the intricate relationship between individual risk factors, social context, and the ultimate outcome of femicide.

### 3.4 Data

The data used for this study were sourced from Ecuador. There are several reasons why Ecuador is an intriguing subject of study. First and foremost, Ecuador’s criminal law underwent a significant transformation in 2014, distinguishing femicide from common murder. This legal distinction provides a unique opportunity to analyze the effectiveness of such legislative changes in combating gender-based violence.

Secondly, Ecuadorian judicial authorities have been diligently collecting statistics on femicides and female violent deaths since that pivotal year. This data availability enables a comprehensive analysis of the trends and patterns surrounding femicide cases, shedding light on the prevalence and characteristics of these crimes. Furthermore, it is essential to consider the broader socio-economic and cultural context of Ecuador (Shai et al., 2022) suggested that violence against women tends to be more persistent in developing countries like Ecuador when compared to their developed counterparts. Therefore, understanding the drivers of femicide in a developing country such as Ecuador may reveal insights and nuances that differ from the existing body of research primarily focused on developed nations.

### 3.5 Data description

Our analysis draws upon a comprehensive dataset derived from the inaugural National Survey on Family Relations and Gender Violence against Women in Ecuador (ENVIGMU), conducted by the National Institute of Statistics and Census (INEC) in 2011 and Consejo de la Judicatura in 2022. Our data comprises 1,325 observations of criminal cases, which include both femicides and violent deaths of women in the 24 provinces of Ecuador over the 2014–2022 period. The distinction between these two crimes was determined by the Judiciary system in accordance with the current criminal law, effective from 2014.

Furthermore, these official records include the date and location of these crimes and information about the victim and the perpetrator such as their age, education, marital status, among others. This information was collected by judicial officers and the police force throughout the formulation and resolution of the cases, and it was later compiled into a single record by the Institute of Statistics and Census.

The characteristics of this dataset allow for examining patterns of femicide including geographical disparities, responses, and victim and perpetrator characteristics, between 2014 and 2021.

Ecuador, like many other countries, grapples with limited data on gender violence. According to the ENVIGMU survey, approximately 25% of women in Ecuador have experienced some form of sexual violence. This survey also reveals 8.7% who reported being kissed or touched against their will, 4.1% forced to disrobe to reveal their private parts, and 3.5% compelled to witness the private parts of others, even if they were minors.

The survey also reveals that a significant proportion of young women who endured sexual violence had close relationships with their perpetrators as the case of Turkey (Toprak and Ersoy, 2017), including fathers (3.5%), stepfathers (5.8%), and brothers (4.4%), underscoring the deeply rooted nature of the problem.

Between August 10, 2014, and February 6, 2022, Ecuador recorded a staggering total of 1,325 femicides and violent deaths of women. These incidents encompass various forms of violent demise, including murders (51.55%), femicides (39.47%), homicides (7.77%), and hired killings (1.21%). Shockingly, this grim tally also includes 144 girls and adolescents under the age of 18, with 35 of them aged between 1 and 4 years old.

Analysis of the data from de la Judicatura (2022) paints a bleak picture of where these crimes occur. Approximately 45.43% of cases took place in locations other than the victim’s home, while 26.94% occurred within the victim’s residence, indicating a higher likelihood of familial involvement in these instances. Moreover, the statistics reveal that 59.62% of these crimes transpired during daylight hours, with 40.38% transpiring at night, particularly during the early morning hours.

**Figure 1:**
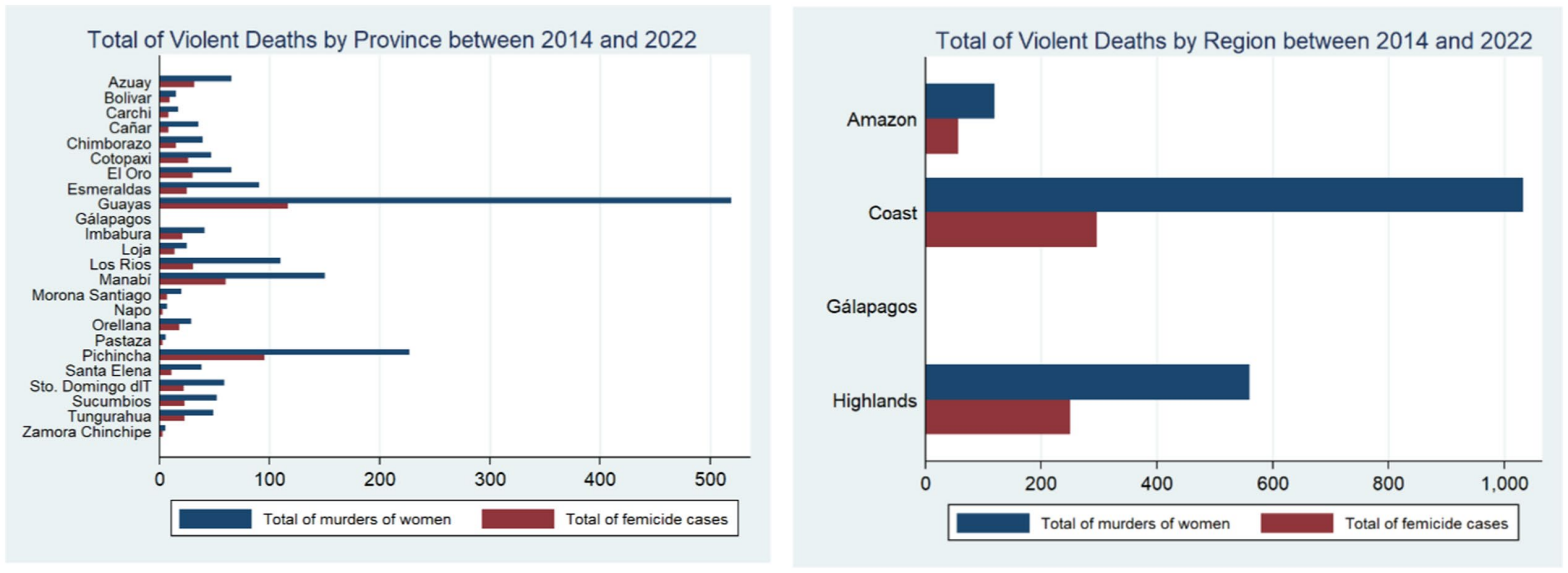
Total of violent deaths by province and region between 2014 and 2022. Left panel represents the total murders of women and femicide cases by province between 2014 and 2022. The right panel illustrates the same information grouped into geographic regions. These indicators were constructed using information from the 2022 National Survey on Family Relations and Gender Violence against Women in Ecuador (ENVIGMU).

A territorial analysis highlights the Coastal region with the highest number of reported crimes. The left panel from Figure 1, for example, shows that this geographic area accounted for roughly 54.64% of femicides and violent deaths. Moreover, the right panel from Figure 1 shows that the province of Guayas, belonging to this geographical zone, represented the majority of reported cases, accounting for about 26.6% of the murders of women.

This region appears to stand out as an epicenter of lethal violence against women in Ecuador. However, it is important to highlight that the Coastal region has been the most densely populated area across time. In 2022, for example, this region represented approximately 50% of the women population. Thus, accounting for the population distribution is essential to better understand the geographical patterns of violence.

**Figure 2:**
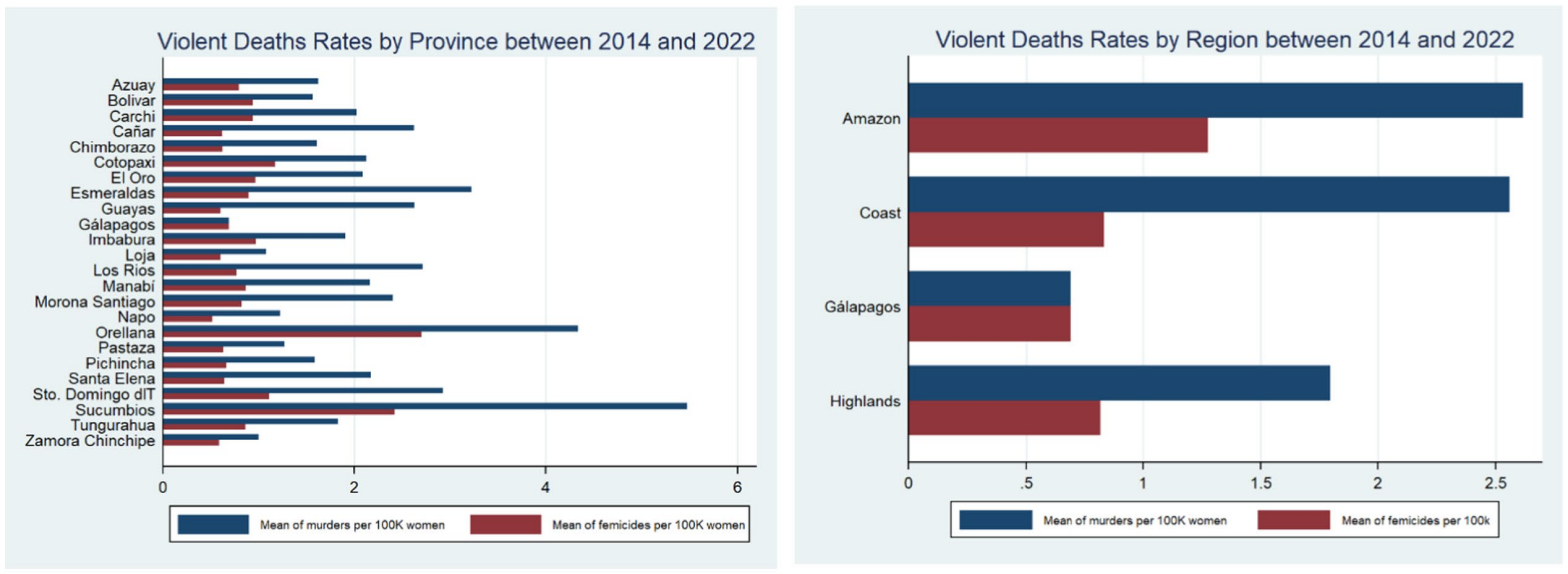
Violent deaths rates by province and region between 2014 and 2022. The left panel represents the average number of murders and femicide cases per 100,000 women by province over the 2014–2022 period. The right panel illustrates the same information but grouped into geographic regions. These rates were constructed using official records. The information about the violent deaths and femicide cases stems from the 2022 National Survey on Family Relations and Gender Violence against Women in Ecuador (ENVIGMU) whereas data on the female population was obtained from the 2023 Statistical Report about Femicide generated by the Ministry of Women.

A per capita analysis depicts the Amazon region as the most violent against women. Figure 2, for instance, shows that between 2014 and 2022, Orellana and Sucumbios were the two provinces with the highest incidence of violent deaths with an average of 4.33 and 5.47 murders per 100,000 women, respectively. These two provinces also had the highest femicide rates over this period, with 2.70 and 2.40, respectively.

Intriguingly, only 5.20% of victims reported any previous instances of violence by their aggressors, leaving a substantial 63.77% of perpetrators unidentified. Among those identified 12% were partners or cohabitants, while the remaining 24.23% included ex-partners, friends, acquaintances, and non-family members, emphasizing the dire need for a comprehensive protection system for victims.

Characteristics of Victims and Perpetrators: The victims’ profile reveals that the majority fall within the age range of 25 to 34 years, with elementary education being the most common educational level. A significant percentage of victims were single (61.96%), followed by married individuals (18.94%). Mestizo women, that is women of mixed Indigenous and European ancestry, accounted for 85.89% of the victims, with 5.35% identifying as Indigenous. Most victims were engaged in some form of occupation, including domestic work, farming, or trade, among others. Furthermore, 55.09% of female victims had at least one child.

Turning to the perpetrators, a quarter of them fell within the 25 to 34 age group, with high school being the predominant level of education. A majority were single (53.66%), followed by divorced (29.66%), and married (15%). Mestizo was the most common self-identification among perpetrators (89.66%), with 6% identifying as Afro-Ecuadorian. In terms of occupation, 69.13% were employed in various roles, such as farming, day labor, trade, or construction. Alarmingly, 19% of perpetrators had multiple criminal records, including charges for robbery, murder, possession of weapons, sexual crimes, and intimidation. Perpetrators in the commission of these crimes frequently used firearms, bladed weapons, and blunt objects.

Alarming Trends in 2021: Figure 3 shows the total number of violent deaths and femicides by year over the 2014–2022 period. The year 2017 concentrated the highest number of femicides, 101 cases, and represented roughly 16.69% of all the cases. The year 2022 was the second most violent, accounting for 89 femicides or 14.71% of all the cases. Moreover, this year also exhibited the highest number of killings of women, 422 cases. This figure represented about a quarter of all deaths and an 87% increase from the 226 cases reported over the last year.

**Figure 3:**
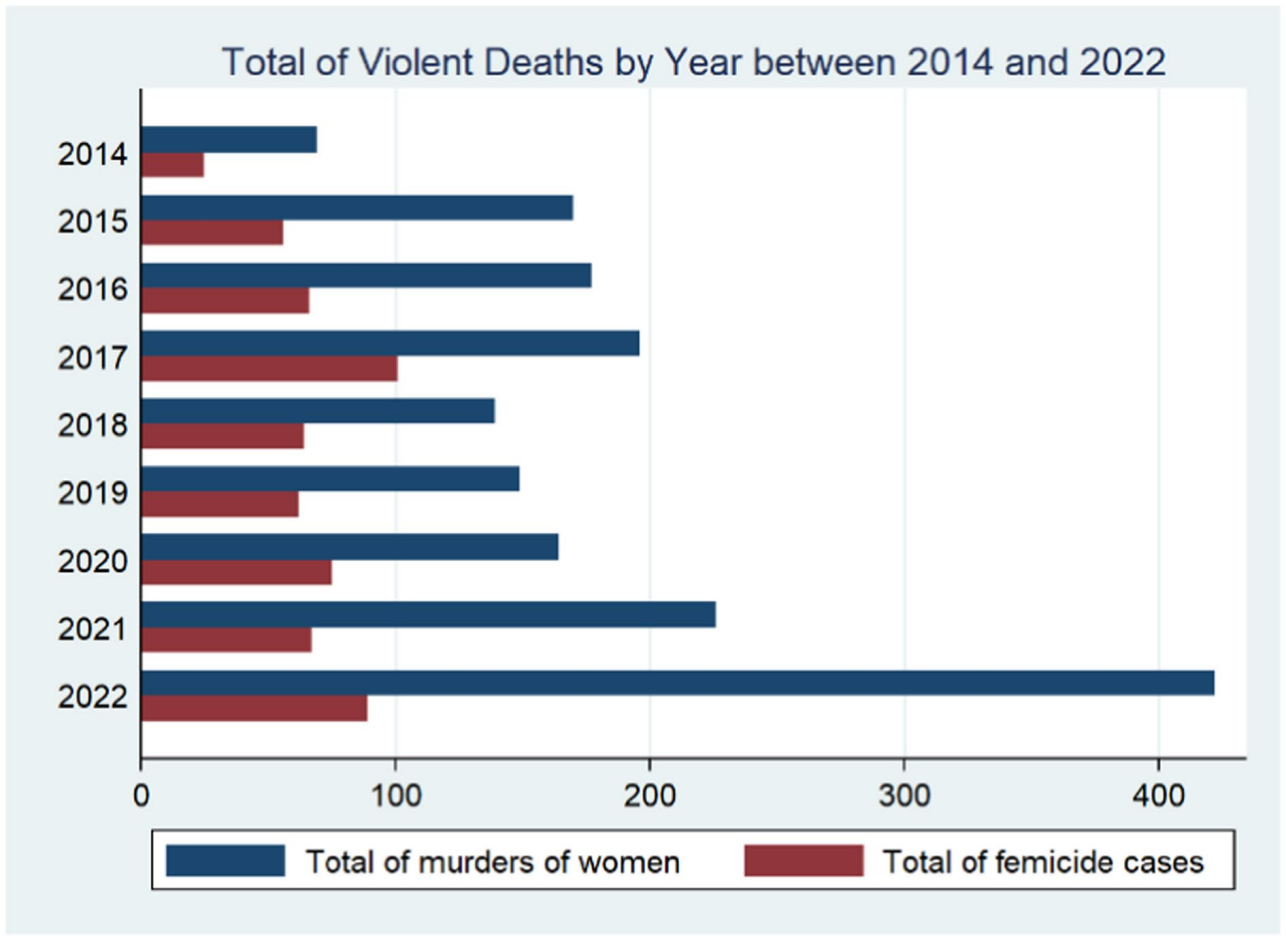
Violent deaths rates by province and region between 2014 and 2022. Figure 2 represents the total murders of women and femicide cases by year between 2014 and 2022. These indicators were constructed using information from the 2022 National Survey on Family Relations and Gender Violence against Women in Ecuador (ENVIGMU).

**Figure 4:**
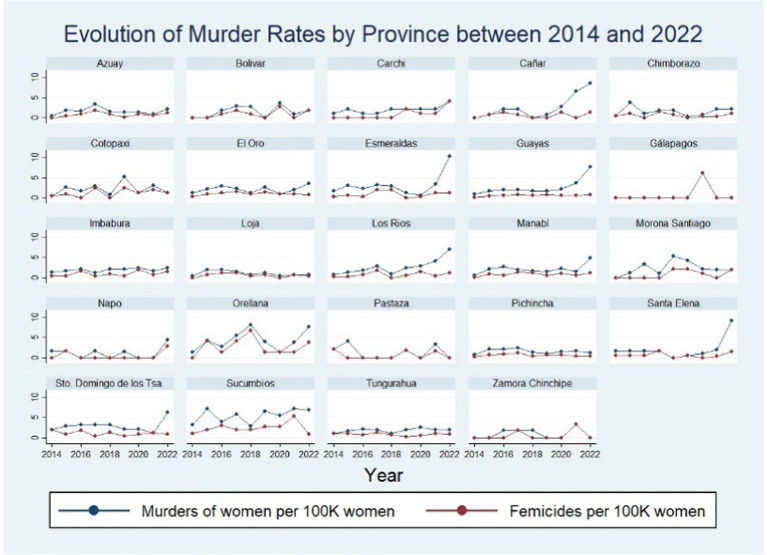
Evolution of murder rates by province between 2014 and 2022. Illustrates the evolution of murders of women and femicide cases by 100,000 over the 2014–2022 period. These rates were constructed using official records. The information about the violent deaths and femicide cases stems from the 2022 National Survey on Family Relations and Gender Violence against Women in Ecuador (ENVIGMU) whereas data on the female population was obtained from the 2023 Statistical Report about Femicide generated by the Ministry of Women.

Figure 4 shows the evolution of the murder rates by province between 2014 and 2022. The murder rates by province in Ecuador oscillated below five killings per 100,000 women except in Cañar, Esmeraldas, Guayas, Los Ríos, Orellana, Santo Domingo, Santa Elena, and Sucumbios. Moreover, three of these provinces, all belonging to the Coast region experienced a substantial surge in violence in 2022, namely Esmeraldas, Santa Elena, and Guayas. Esmeraldas, for example, experienced a roughly seven-unit increase in its murder rate from 3.43 to 10.38 killings per 100,000 women between 2021 and 2022.

Guayas also experienced a big surge of violence in 2022, reaching roughly 7.73 murders per 100,000 women, a four-unit increment or a 110% increase from the previous year. It is crucial to note that these alarming levels of violence also led to the city of Guayaquil, Guayas capital, being ranked among the 50 most violent cities in the world, as reported by the Consejo Ciudadano para la Seguridad Pública y la Justicia Penal A.C. (2022). This ranking was attributed to rising murder rates, robberies, and drug-related incidents, adding to the complex tapestry of violence in Ecuador.

On the other hand, two provinces in the Amazon, Orellana and Sucumbios, exhibited the highest femicide rates between 2014 and 2022. The average rates in these two provinces surpassed five murders per 100,000 women while femicide rates for the rest of the provinces tended to remain below three cases. Moreover, Orellana showed a significant surge in femicides in 2018, reaching a rate of about 6.71 cases, while Sucumbios experienced approximately 5.38 femicides per 100,000 women in 2021.

### 3.6 Variable description

Our dependent variable is a dichotomous variable that takes the value of one if a woman was a victim of femicide and zero if she was murdered in any other violent way. Given our data comes from official records, we rely on Ecuador’s legal framework to distinguish between a femicide and a murder. Regarding independent variables, Table 2 presents a comprehensive overview of the variables crucial to our study on femicide in Ecuador. These variables have been selected based on their relevance and potential impact on understanding the underlying drivers of this issue. Additionally, we have provided essential statistical insights into these variables, offering a foundational understanding of the data, whichwasexplored in the previous section of our research.

**Table 2:** Variables included in the empirical model as candidate determinants of femicide in Ecuador.

*Determinant*	*Variables*	*Data source*
(A) Main characteristics		Femicidios Ec. Consejo de la Judicaturahttps://www.funcionjudicial.gob.ec/femicidiosec
(A1) Victim and perpetrator ages	Victim age and Perpetrator age	
(A2) Victim and perpetrator ethnic self-identifications	Categorical variable: Afro-Ecuadorian, White, Indigenous, Other and whose reference category is the mestizo self-identification of the victim and the perpetrator.	
(A3) Victim and perpetrator civil statuses	Categorical variable: union free, divorced, single, widowed and whose reference category is the married marital status of the victim and the offender.	
(A4) Victim and perpetrator professions	Dummy variable that takes the value of 1 if the victim or offender had some type of profession.	
(A5) Victim and perpetrator nationalities	Categorical variable: Colombian, Venezuelan and whose reference category is the Ecuadorian nationality of the victim and the perpetrator.	
(A6) Victim and perpetrator level of education	Categorical variable: High school education, higher education and whose reference category is the basic education of the victim and the offender.	
(A7) Victim-aggressor relationship	Categorical variable: partner, former cohabitant, family member, friend, work relationship, acquaintance, unknown and whose reference category is cohabitant of the victim.	
(B) Social disruption		
(B2) Area	Dummy variable that takes the value of 1 if the victim lives in a rural area.	
(C) Presence of risk factor		
(C1) Cause of death	Categorical variable: Strangulation, stab wound, gunshot wound, contusion injury, asphyxia, others, and whose reference category is hanging of the victim.	

To provide a comprehensive overview of the dataset we have been examining, Tables 3, 4 offer concise summaries of the quantitative and qualitative variables discussed earlier in our research. These tables encapsulate the essential descriptive statistics that underpin our analysis, allowing for a quick reference to key findings and trends within the data.

**Table 3:** Descriptive statistics of quantitative variables.

Variable	Mean	Q1	Median	Q3	Std. dev.	Min.	Max.
Victim Age	33.58	25	28	41	15.312	1	98
Number of the victim’s children	1.66	1	2	2	1.016	0	7
Perpetrator Age	33.85	30	34	34	9.103	13	85

**Table 4:** Descriptive statistics of qualitative variables.

Variable/categories	Freq. (%)	Variable/categories	Freq. (%)
Victim’s ethnic self-identification		Perpetrator’s ethnic self-identification	
Mestizo	85.89	Mestizo	89.66
Afro-Ecuadorian	4.23	Afro-Ecuadorian	6.04
White	2.79	White	0.68
Indigenous	5.36	Indigenous	2.57
Other	1.74	Other	1.06
Victim’s level of education		Perpetrator’s level of education	
Basic	49.28	Basic	30.87
High school	39.77	High school	64.91
University	5.06	University	2.04
None	5.89	None	2.19
Victim’s civil status		Perpetrator’s nationality	
Married	18.94	Ecuadorian	96.30
Free union	2.72	Colombian	1.28
Divorced	12.83	Venezuelan	1.66
Single	61.96	Other	0.75
Widowed	3.55	Perpetrator’s occupation	
Victim’s profession		Yes	88.91
Yes	88.91	No	11.09
No	11.09	Perpetrator on most wanted list	
Victim was pregnant		Yes	0.98
Yes	73.74	No	99.02
No	26.26	Perpetrator arrest order	
Victim has a disability		Yes	6.72
Yes	3.40	No	93.28
No	96.60	Other perpetrators	
Victim’s nationality		Yes	10.57
Ecuadorian	95.17	No	89.43
Colombian	1.81	Perpetrator’s profession	
Venezuelan	1.43	Yes	76.98
Other	1.58	No	23.02
Cause of victim’s death		Fugitive perpetrator	
Hanging	2.72	Yes	7.77
Strangulation	13.81	No	92.23
Stab wound	33.81	Infraction perpetrator	
Gunshot wound	29.89	Yes	97.21
Contusion injury	11.17	No	2.79
Asphyxia	4.30	Antecedents’ perpetrator	
Other	4.30	Yes	19.02
Victim-aggressor relationship		No	80.98
Spouse, cohabitant	23.70	Perpetrator used type of weapon	
Partner	6.42	Yes	99.70
Former cohabitant	9.36	No	0.30
Family member	36.53	Perpetrator years of deprivation	
Friend	2.64	Yes	33.28
Work relationship	0.45	No	66.72
Other relatives	2.94	Crime scene	
Acquaintance	9.51	Victim’s domicile	26.94
Unknown	8.45	Perpetrator’s domicile	2.04
Perpetrator’s civil status		Family member’s domicile	20.75
Married	14.94	Another domicile	4.83
Free union	1.13	Another place	45.43
Divorced	29.58	Area of residence	
Single	53.66	Urban	71.70
Widowed	0.68	Rural	28.30

## 4 Methodological approach

### 4.1 Ethics statement

Ethical concerns about secondary analysis of a publicly available dataset on violence usually revolve around potential harm to individual subjects. This risk is significantly larger if the dataset has identifying information or has not been appropriately de-identified (Fielding and Fielding, 2003; Tripathy, 2013).

For our analysis, the data on femicides and violent murders of women is publicly available on the Attorney General’s Office website: https://www.fiscalia.gob.ec/analitica-muertes-de-mujeres-en-contexto-delictivo/. However, only de-identified data was accessed by our research team; thus, the current study does not pose a threat to the confidentiality of the victims’ information. Moreover, since our research does not include any interaction or intervention with human subjects nor includes any access to identifiable private information, we did not seek an International Board Review exemption.

### 4.2 The model

Considering the inherent characteristics of our dataset, binary choice models present a fitting approach for our empirical analysis. These models allow us to effectively control for the myriad factors that impact the likelihood of a woman becoming a victim of femicide. Our primary objective in adopting this method is twofold: firstly, to discern the presence of statistically significant influences, and secondly, to ascertain the direction and magnitude of their effects.

Our analytical framework encompasses a Probit regression model designed to elucidate the probability of a woman falling victim to femicide in Ecuador. In this regard, we put forth the following Probit regression showed in Equation 1:


(1) $$\text{Femicid}e_{i}=\int_{-\infty }^{\varphi +\beta X_{i}+\mu_{i}}\frac{1}{\sqrt{2\pi }}e^{\left[-\frac{t^{2}}{2}\right]}dt$$

Where $\text{Femicid}e_{i}$ is our dependent dichotomous variable that takes the value of 1 if a woman *i* has been a victim of femicide or 0 if she was murdered in any other violent way; $X_{i}$ is our set of control variables that include characteristics related to the victim, the perpetrator and the macroenvironment and $\mu_{i}$ is the stochastic error term of the model.

Our independent variables adhere to a typical approach, involving the characterization of the victim, the perpetrator, and the homicide itself. Specifically, we include variables that capture crucial information about these three facets. For the victims, our control variables encompass age, level of education, marital status, ethnicity, occupation, nationality, presence of any disabilities, cause of death, pregnancy status, and the number of children they had.

In characterizing the perpetrators, we incorporate variables that account for their age, level of education, marital status, ethnicity, occupation, nationality, their relationship with the victim, the type of weapon used to commit the crime, their response following the crime (such as surrendering to authorities, fleeing, or dying by suicide), and any prior criminal record, complaints, or criminal accusations.

Furthermore, we endeavor to capture environmental factors tied to the location of the crime. Therefore, we include variables such as province and the categorization of the area as urban or rural. This comprehensive set of variables ensures that our empirical analysis is robust and capable of shedding light on the multifaceted factors associated with femicide in Ecuador. Moreover, four models we run to test four hypotheses.

#### 4.2.1 Model 1: perpetrator characteristics

This model tests the hypothesis that the individual characteristics of perpetrators are significant predictors of femicide. We hypothesize that certain attributes, such as age, education level, and prior criminal history, influence the likelihood of committing femicide. Understanding the impact of perpetrator characteristics alone helps isolate their specific contributions to femicide, which is essential for targeted interventions.

#### 4.2.2 Model 2: victim characteristics

The second model only considers victim characteristics. This model examines the hypothesis that victim characteristics are significant determinants of femicide. We hypothesize that factors such as education level and occupation influence the probability of a woman’s death being classified as femicide. Focusing on victim characteristics provides insights into how specific attributes may increase vulnerability to femicide, aiding in protective measures.

#### 4.2.3 Model 3: victim and perpetrator characteristics

The third model combines characteristics of both perpetrator and victim to test the combined influence on femicide likelihood. We hypothesize that the interaction between these factors provides a more comprehensive understanding of femicide, since combining these characteristics allows us to explore how their interaction affects femicide, offering a holistic view of the problem.

#### 4.2.4 Model 4: COVID-19 influence

Finally, model fourth evaluates the influence of the COVID-19 pandemic on femicide, incorporating both perpetrator and victim characteristics as well as pandemic-related variables. We hypothesize that the pandemic has altered the dynamics of femicide. Assessing the pandemic’s effect helps understand how crises influence femicide rates and informs policy responses during emergencies.

## 5 Results

Table 5 presents our estimation results. Column (1) presents Model 1 that considers only the characteristics of the perpetrator, while column (2) shows the estimates of Model 2 that takes into account the characteristics of the victim. Moreover, column (3) presents the estimates of Model 3, which considers the characteristics of both victim and perpetrator. These three specifications include the characteristics of the place where the crime occurred. Finally, column (4) presents Model 4, which includes data from the most severe period of the COVID-19 pandemic, that is, between 2020 and 2021.

**Table 5:** Estimation results.

*Dep.* Var. *Femicide*	*(1)*	*(2)*	*(3)*	*(4)*
*Gender*				
*Man*	0.814***		0.812***	3.277***
	(0.185)		(0.217)	(0.782)
*Perpetrator’s age*	0.072***		0.004	0.055
	(0.020)		(0.024)	(0.053)
*Perpetrator’s age squared*	−0.001**		0.000	0.000
	(0.000)		(0.000)	(0.001)
*Perpetrator’s level of education (Basic = 0)*				
*High school*	1.046***		0.803***	−0.286
	(0.139)		(0.161)	(0.489)
*University*	1.362***		1.368***	−0.475
	(0.294)		(0.332)	(0.616)
*None*	1.084***		0.978***	-0.085
	(0.117)		(0.136)	(0.387)
*Perpetrator’s civil status (Married = 0)*				
*Single*	-0.163		-0.068	-0.064
	(0.124)		(0.141)	(0.498)
*Divorced*	-0.442		-0.513	0.145
	(0.255)		(0.301)	(0.684)
*Widowed*	-0.579		−1.148	
	(0.536)		(0.640)	
*Perpetrator ethnic self-identification (Mestizo = 0)*				
*Afro-Ecuadorian*	−0.271		−0.195	−0.863
	(0.182)		(0.212)	(0.480)
*White*	0.137		0.163	1.450
	(0.344)		(0.388)	(0.863)
*Indigenous*	−0.272		−0.427	
	(0.505)		(0.648)	
*Other*	−0.456		−0.387	0.662
	(0.193)		(0.202)	(0.868)
*Perpetrator’s nationality (Ecuadorian = 0)*	0.128		0.205	−0.611
	(0.111)		(0.125)	(0.312)
*Victim age*		0.071***	0.065***	0.082**
		(0.013)	(0.017)	(0.027)
*Victim age^2^*		−0.001***	−0.001***	−0.002***
		(0.000)	(0.000)	(0.000)
*Victim’s level of education (None = 0)*				
*High school*		−0.099	−0.246	−0.382
		(0.101)	(0.127)	(0.327)
*University*		−0.677**	−0.686*	−0.052
		(0.214)	(0.272)	(0.587)
*Basic*		−0.912***	−0.792***	−0.901*
		(0.144)	(0.180)	(0.444)
*Victim’s civil status (Married = 0)*				
*Single*		0.033	0.146	−0.150
		(0.103)	(0.132)	(0.284)
*Divorced*		−0.217	−0.062	−0.324
		(0.189)	(0.250)	(0.455)
*Widowed*		0.468	0.522	1.335
		(0.307)	(0.340)	(0.695)
*Victim ethnic self-identification (Mestizo = 0)*				
*Afro-Ecuadorian*		−0.278	−0.036	0.138
		(0.201)	(0.206)	(0.486)
*White*		−0.158	−0.098	−0.869
		(0.208)	(0.237)	(0.630)
*Indigenous*		0.148	0.359	−0.212
		(0.220)	(0.305)	(0.571)
*Other*		0.236	0.283	−0.847
		(0.262)	(0.222)	−1.143
*Perpetrator’s nationality (Ecuadorian = 0)*		0.142	−0.224	0.840
		(0.198)	(0.233)	(0.506)
*Antecedents’ perpetrator (Yes = 1)*	0.010		0.030	−0.452
	(0.110)		(0.121)	(0.298)
*Perpetrator used type of weapon: (Edged Weapon = 0)*				
*Firearm*	−0.443***		−0.593***	−1.654***
	(0.124)		(0.135)	(0.324)
*Strangulation Device*	0.403*		0.498**	−0.314
	(0.166)		(0.187)	(0.426)
*Substances*	−0.567		−0.527	
	(0.367)		(0.399)	(0.000)
*Other*	−0.013		0.238	−0.264
	(0.124)		(0.145)	(0.317)
*Perpetrator on most wanted list (Yes = 1)*	0.610		0.668*	0.292
	(0.409)		(0.341)	(0.599)
*Perpetrator arrest order (Yes = 1)*	0.427*		0.297	−0.940
	(0.217)		(0.234)	(0.588)
*Other perpetrators (Yes = 1)*	−1.554***		−1.666***	−1.208***
	(0.193)		(0.215)	(0.367)
*Perpetrator’s profession (Yes = 1)*	0.190		0.136	0.273
	(0.150)		(0.159)	(0.364)
*Perpetrator is a fugitive (Yes = 1)*	0.162		−0.058	0.706
	(0.213)		(0.222)	(0.465)
*Perpetrator was in rehab. center (Yes = 1)*	0.396*		0.349	1.140**
	(0.192)		(0.199)	(0.419)
*Perpetrator has a history of violence (Yes = 1)*		−0.841***	−0.202	1.023**
		(0.204)	(0.243)	(0.395)
*Victim has disability (Yes = 1)*		−0.847**	−1.096**	
		(0.272)	(0.346)	
*Victim was pregnant (Pregnant = 1)*		0.306	0.236	
		(0.248)	(0.304)	
*Victim has children (Pregnant = 1)*		0.498***	0.476***	0.130
		(0.095)	(0.117)	(0.259)
*Victim’s profession (Yes = 1)*		0.227*	0.318*	0.015
		(0.111)	(0.135)	(0.379)
*Other victims (Yes = 1)*		0.039	−0.349**	
		(0.109)	(0.132)	
*Covid (years 2020–2021 = 1)*			0.383**	
			(0.132)	
*Crime location: (Perpetrator residence = 1)*				
*Victim’s residence*	−0.391	−0.752**	−0.251	−0.362
	(0.288)	(0.266)	(0.287)	(0.668)
*Family residence*	0.096	0.040	−0.015	0.510
	(0.291)	(0.271)	(0.291)	(0.645)
*Another residence*	−0.628	−1.221***	−0.900**	−1.240
	(0.343)	(0.320)	(0.343)	(0.706)
*Another location*	−0.398	−1.214***	−0.603*	−1.125
	(0.285)	(0.261)	(0.281)	(0.632)
*Area of residence (Rural = 1)*				
*Urban*	−0.215	−0.262**	−0.306**	−0.777***
	(0.095)	(0.088)	(0.107)	(0.223)
*Region: (Coast = 0)*				
*Highlands*	−0.329	−0.848	−0.059	0.397
	(0.471)	(0.526)	(0.507)	(0.619)
*Amazon Region*	0.082	0.503***	0.210	0.115
	(0.099)	(0.087)	(0.113)	(0.248)
*Constant*	−2.452	−0.480	−1.836**	−3.081
	(0.522)	(0.400)	(0.623)	−1.579
*chi2*	462.707	372.308	498.297	243.465
*N*	1,320	1,320	1,320	371
*Aic*	1046.049	1276.899	896.21	243.498
*Bic*	1222.352	1416.904	1176.221	427.559
*Rank*	34	27	54	47
*% correctly predicted*	0.8462	0.7871	0.878	0.9084
*ROC curve*	0.9093	0.8535	0.9426	0.9745

*p < 0.05; **p < 0.01; ***p < 0.001.

The econometric models employed in this study illuminate the factors that influence the occurrence of femicide in Ecuador. These models demonstrate a commendable overall fit, evident through significant Chi-squared statistics and high correct classification rates exceeding 78%. The findings derived from our regression models are outlined below.

### 5.1 Perpetrator characteristics

The gender of the perpetrator, particularly male, emerges as a significant and positive determinant of femicide, indicating that being male substantially elevates the likelihood of committing femicide. Furthermore, the perpetrator’s age exhibits a U-shaped relationship with femicide. An increase in the perpetrator’s age is initially associated with an escalated probability of becoming a femicide perpetrator. However, beyond a certain threshold, this probability diminishes.

Education levels of the perpetrator consistently demonstrate significance and a positive association with femicide across all models, except when pandemic-related data is included. This suggests that regardless of the level of education, higher educational attainment heightens the probability of perpetrating femicide.

The marital status of both the perpetrator and the victim does not emerge as a significant determinant. Similarly, ethnic self-identification and the perpetrator’s criminal history, as indicated by prior criminal records, do not exhibit significance in femicide. Interestingly, the use of firearms as a murder weapon emerges as a significant and negative factor, implying that an increased utilization of firearms diminishes the likelihood of the crime being classified as femicide.

Notably, being on the most wanted list in Ecuador demonstrates a positive and significant coefficient in Model 3, suggesting that it elevates the probability of femicide. Additionally, having an outstanding arrest warrant is significant in Model 1, indicating that it augments the likelihood of the crime being femicide.

The presence of other perpetrators involved in the crime is linked to a reduction in the likelihood of femicide. Moreover, being on probation is significant in Models 1 and 4, indicating that it heightens the probability of the crime being categorized as femicide. The perpetrator’s history of violence yields diverse outcomes - Model 2 exhibits a negative and significant coefficient, suggesting that prior violent behavior reduces the likelihood of femicide. However, Model 4, takes on a positive and significant character, indicating that during the pandemic, a history of violence increases the probability of femicide.

### 5.2 Victim characteristics

The education level of the victim emerges as a negative and significant factor, particularly in the highest education category, except in Models 3 and 4. This signifies that having any education reduces the probability of being a femicide victim. The pregnancy status of the victim does not demonstrate significance. However, having children or a profession is associated with an increased likelihood of the crime being classified as femicide, as indicated by positive and significant coefficients in Models 2 and 3. Notably, this effect is not observed in Model 4. Additionally, the presence of other victims decreases the likelihood of femicide in Model 3, as suggested by a negative and significant coefficient.

During the pandemic, crimes were more likely to be categorized as femicides, as demonstrated by the positive and significant coefficient of the “pandemic” variable in Model 3. On the other hand, having a disability is associated with a reduced probability of a woman experiencing this type of violence, as reflected in the significant and negative coefficients observed in Models 2 and 3.

### 5.3 Crime-related variables

The location of the crime significantly influences the likelihood of femicide. Crimes occurring in the victim’s residence, another residence, or another location are less likely to be classified as femicides, as indicated by negative and significant coefficients in Models 2 and 3. Urban areas are less likely to witness femicides, as evidenced by the significance of the “urban area” variable in Models 2, 3, and 4. In Model 2, only the Amazonia region emerges as a significant determinant, suggesting that crimes occurring in this region have a higher probability of being classified as femicides.

These results offer a comprehensive understanding of the intricate determinants of femicide in Ecuador, encompassing a multitude of perpetrator, victim, and crime-related characteristics. They provide a valuable foundation for the development of targeted policies and interventions aimed at preventing femicide within the country.

## 6 Discussion

This research aimed to uncover the determinants and factors contributing to femicide in the context of Ecuador’s emerging economy. Femicide, as a form of gender-based violence, represents a critical issue that extends beyond social concerns and has economic implications.

The empirical analysis revealed valuable insights into the variables impacting this likelihood, allowing us to discard certain factors as irrelevant in the Ecuadorian context. From our examination of 1,325 violent deaths of women occurring in Ecuador between September 2014 and February 2022, we observed that 523 of these cases (39.5%) met the criteria for femicide, while the remaining 802 cases (60.5%) were attributed to other forms of violent incidents.

The aforementioned statistics underscore that women are at a heightened risk of experiencing mistreatment at the hands of their aggressors, who often manifest cruelty as a manifestation of misogyny. This cruel treatment reduces women to the status of objects, to be utilized and ultimately discarded (Lemus et al., 2010; Carcedo and Sagot, 2000).

Femicide results in direct and indirect economic costs for a country, including the loss of human capital, reduced labor force participation, increased healthcare expenses, and a burden on the legal and judicial systems (Toprak and Ersoy, 2017). Understanding the determinants of femicide is essential for constructing targeted policies and interventions that can mitigate its impact on society.

Our analysis uncovers the intricate interplay of various factors that increase the likelihood of femicide. Education, employment status, and geographical location emerge as crucial determinants in femicide. Notably, the perpetrator’s education, employment status, and the location of the crime exhibit robust correlations with femicide incidents, emphasizing their pivotal roles in understanding the occurrence of this crime.

A deeper exploration into the mechanisms through which education influences violence against women is warranted. The consistent positive association between the level of aggressor’s education and femicide, regardless of educational attainment, raises questions about the role of education in shaping attitudes and behaviors (Hernández, 2021). This finding necessitates a more comprehensive understanding of how education can serve as a preventive measure against gender-based violence.

The consistent positive association between the level of aggressor’s education and femicide, regardless of educational attainment, raises questions about the role of education in shaping attitudes and behaviors (Hernández, 2021). This finding necessitates a deeper exploration into the mechanisms through which education influences violence against women.

The significance of being on the most wanted list and having an outstanding arrest warrant in the context of femicide is an important revelation. This indicates that law enforcement agencies play a crucial role in tracking and preventing femicide. These findings emphasize the importance of effective criminal justice responses to deter potential perpetrators.

The intriguing reversal in the effect of a perpetrator’s history of violence, depending on the presence of a pandemic, warrants further investigation. During crises, pre-existing violent tendencies seem to become more pronounced, potentially indicating heightened stress and tensions.

The negative relationship between the education level of the victim and femicide is an interesting observation. This suggests that education may serve as a protective factor for potential victims, possibly by providing them with greater agency and resources to avoid dangerous situations (Hernández, 2021).

The presence of children or a profession as a risk factor for femicide, particularly in specific models, merits consideration. It highlights the need for holistic support structures that ensure the safety of women who fulfill caregiving roles or engage in professional activities (Toprak and Ersoy, 2017; Gould, 2022; Atuk and Craddock, 2023).

The location of the crime is a pivotal factor in understanding femicide in Ecuador. Notably, crimes occurring in domestic settings, such as the victim’s residence, are associated with a lower likelihood of the crime being a femicide. These finding sheds light on the complex dynamics within domestic environments, suggesting that while domestic settings may witness severe forms of violence, they are less likely to escalate to the extreme of femicide (Cullen et al., 2019).

However, this lower incidence of femicides at home by no means diminishes the significance of addressing domestic violence. Instead, it emphasizes the importance of tailored interventions to prevent the progression from domestic violence to femicide, and the need for comprehensive support systems to ensure the safety and well-being of potential victims within their own homes.

Moreover, the location of the crime played a pivotal role, with femicides being more likely to occur in rural areas. This association underscores the economic disparities and social challenges often faced by rural populations (Swanson, 2013). Policymakers should prioritize economic development in rural areas, creating jobs and educational opportunities to reduce femicide incidence.

The regional variation in femicide, with the Amazonia region being particularly significant, underscores the importance of understanding regional disparities in violence and tailoring interventions to specific geographic contexts. The prevalence of the rural population in the Amazon may be a key factor to understand these disparities. For example, roughly 37% of Ecuadorian households live in rural areas whereas in the Amazon this proportion surpassed the 60%.

The previous finding is in line with the crime opportunity theories. Felson and Clarke (1998), for instance, explains that crime is not a random event but the result of the circumstances, particularly 3 factors: a victim, a motivated aggressor, and an absence of control. Historically, rural areas in Ecuador have been characterized by the absence of crime-control strategies, limited presence of the judiciary, and lack of access to services like health or education; thus, not only is it understandable why these geographic regions exhibit a high incidence of femicide, but this reality also demonstrates the urgency of policy intervention. However, it is important to emphasize that these findings may be a consequence that more disenfranchised and marginalized portions of the population, as is the case of the Amazon, are policed differently.

Intriguingly, the pandemic variable’s positive coefficient in Model 3 suggests that the COVID-19 pandemic has had a significant impact on the likelihood of femicide. This result calls for a deeper exploration of the pandemic’s influence on domestic violence and gender-based violence more broadly (Morena et al., 2022).

From a public policy perspective, our results shed some light on femicide in Ecuador. Considering that education, specifically in the perpetrator, is a deterrence factor of the probability of committing femicide, information campaigns and educational programs in gender equality and policies of reduction of violence addressed to men in early stages of educational development can become a priority for the Ecuadorian government. This involves implementing comprehensive and inclusive education on gender-based violence, gender inequality, gender bias, and machismo, starting from kindergarten and primary schools. Engaging children in strength-based learning that models gender-affirming, inclusive, equal, and respectful ways of being to oneself and others can create a foundation for long-term cultural change.

In addition, there is no doubt that gender inequality (Reckdenwald et al., 2018), discrimination (Lund et al., 2020), aggression, and the death of women constitute a violation of human rights and require new measures and laws that protect and protect women, and above all that the full weight of the law is applied to the aggressor. It is recommended not to hide information when reporting this type of crime as it becomes an important factor that prevents judges from classifying the crime as an act other than violent death (Anderson, 2013).

### 6.1 Limitations

While our study has provided valuable insights into the determinants of femicide in Ecuador within an economic framework, it is essential to acknowledge the limitations of our research. First, it is important to recognize that femicide is not an isolated issue and is often interconnected with other serious social and psychological concerns. Suicides following femicides, often referred to as homicide-suicide, may be attributed to psychological stress (Standish and Weil, 2021; Richards et al., 2014). Understanding and addressing the mental health consequences of femicide and the potential triggers for such extreme acts are crucial avenues for future research and policy development, but our study did not delve into these complex psychological dynamics.

Another limitation lies in the psychological impact on children who have lost their mothers and potentially witnessed or experienced violence. While our dataset does not allow for an in-depth examination of this aspect, the work of scholars such as Hardesty et al. (2008) points to the severe psychological consequences faced by these children, emphasizing the need for further research in this domain.

Furthermore, the prevalence of mental health issues among perpetrators of femicide, as highlighted by Cullen et al. (2019) and Hernández (2021), suggests a complex interplay between mental health, violence, and femicide. Future research should delve deeper into this intersection to inform interventions aimed at both preventing femicide and addressing underlying mental health issues. Although our study has provided insights into economic determinants, it does not encompass the full scope of these psychological and social complexities. These limitations should be considered in future research endeavors aimed at comprehensively addressing the issue of femicide in Ecuador.

## 7 Conclusion

In conclusion, the results of this study provide a comprehensive understanding of the multifaceted determinants of femicide in Ecuador. They emphasize the need for a holistic approach that considers both perpetrator and victim characteristics, as well as crime-related variables, in the development of targeted policies and interventions to prevent femicide. Furthermore, the dynamic nature of these determinants, particularly during crises such as the pandemic, underscores the importance of ongoing research and adaptive policy responses to address this issue effectively.

### 7.1 Reflexivity statement

In this study, we have undertaken a rigorous investigation into the determinants of femicide in Ecuador. Given the deeply sensitive and tragic nature of this subject, we acknowledge that our positionality as researchers inevitably shapes how we engage with the topic and interpret the data. We are acutely aware of the socio-political context of gender-based violence, particularly in the context of our backgrounds and biases.

#### 7.1.1 Personal characteristics

Our research team is composed of two women and two men, which we believe provides a balanced perspective on the sensitive issue of gender-based violence. We come from a developing country, where certain behaviors related to violence against women may be more normalized. This context makes us particularly sensitive to issues of gender inequality but also aware of potential biases stemming from cultural norms. Additionally, all team members are from a middle-class socioeconomic background, which may influence our interpretations and perspectives, shaping our understanding of social issues, including gender violence.

#### 7.1.2 Prior experiences and knowledge

As researchers, we bring extensive experience in quantitative methodologies to this study. This includes statistical analysis of large datasets, which allows for a systematic exploration of the determinants of femicide. However, we recognize the limitations of this approach in capturing the full depth of the issue. Qualitative methods, such as in-depth interviews with those close to the victims, could offer richer insights into the socio-cultural dynamics surrounding femicide. Although our primary focus is on quantitative analysis, we are mindful of the value that qualitative approaches could add to our understanding.

#### 7.1.3 Social and political context

This study is situated within a broader socio-political context where gender-based violence remains a critical issue in Ecuador and other developing countries. We are aware of the challenges associated with conducting research in environments where such violence is often underreported and sometimes normalized. To mitigate the influence of these social norms, we made a deliberate effort to ensure a gender-balanced team, which we believe enhances the diversity of perspectives in our analysis.

#### 7.1.4 Potential biases and assumptions

We recognize that our academic training in quantitative analysis may influence our interpretations of the data. There is a risk of distancing ourselves from the lived realities of those affected by gender-based violence, given our reliance on statistical methods. To address this, we have consciously engaged with literature from both quantitative and qualitative paradigms, aiming to remain empathetic and grounded in the socio-cultural realities of the affected individuals. Additionally, the team’s middle-class background may predispose us to certain assumptions about social structures and violence, which we acknowledge as a potential source of bias.

#### 7.1.5 Researcher-participant dynamics

Since this study relies on publicly available data, there were no direct interactions with participants. Nevertheless, we are conscious of the ethical implications of analyzing sensitive data related to femicide. We ensured that all data used in this study were anonymized and respected the confidentiality of both victims and perpetrators, thus adhering to ethical standards in research.

## Data Availability

Publicly available datasets were analyzed in this study. This data can be found here: https://anda.inec.gob.ec/anda/index.php/catalog/919.
